# What Can Cellular Redox, Iron, and Reactive Oxygen Species Suggest About the Mechanisms and Potential Therapy of COVID-19?

**DOI:** 10.3389/fcimb.2020.569709

**Published:** 2020-12-14

**Authors:** Barry B. Muhoberac

**Affiliations:** Department of Chemistry and Chemical Biology, Indiana University-Purdue University Indianapolis, Indianapolis, IN, United States

**Keywords:** COVID-19, redox, ROS—reactive oxygen species, glutathione, ascorbate, iron, chelators, ferroptosis

## Abstract

Accumulating evidence suggests that there are important contributions to coronavirus disease (COVID-19) from redox imbalance and improperly coordinated iron, which cause cellular oxidative damage and stress. Cells have developed elaborate redox-dependent processes to handle and store iron, and their disfunction leads to several serious diseases. Cellular reductants are important as reactive oxygen species (ROS) scavengers and to power enzymatic repair mechanisms, but they also may help generate toxic ROS. These complicated interrelationships are presented in terms of a cellular redox/iron/ROS triad, including ROS generation both at improperly coordinated iron and enzymatically, ROS interconvertibility, cellular signaling and damage, and reductant and iron chelator concentration-dependent effects. This perspective provides the rational necessary to strongly suggest that COVID-19 disrupts this interdependent triad, producing a substantial contribution to the ROS load, which causes direct ROS-induced protein and phospholipid damage, taxes cellular resources and repair mechanisms, and alters cellular signaling, especially in the more critical acute respiratory distress syndrome (ARDS) phase of the infection. Specific suggestions for therapeutic interventions using reductants and chelators that may help treat COVID-19 are discussed.

## Introduction

Hematological and clinical descriptions of patients with coronavirus disease 2019 (COVID-19) are beginning to accumulate (Cao et al., 2020; Zhou et al., 2020) and will, in this perspective, be interpreted and correlated to suggest mechanisms that may in part explain the negative biochemical and physiological changes that are being induced. There is clear evidence of elevated plasma ferritin, decreased hemoglobin, and altered erythrocyte distribution and shape in COVID-19 (Zhou et al., 2020), which together can be interpreted as alteration in iron utilization and handling. Concurrent are the descriptions of acute respiratory distress syndrome (ARDS), “high altitude sickness,” increased lactate, and the use of ventilators to supplement oxygenation. Also observed is greatly elevated C-reactive protein (CRP), implying an inflammatory response that is potentially linked to reactive oxygen species (ROS) oversignaling and ROS-produced cellular damage. In COVID-19, there is increased survival associated with higher selenium level and non-elevated (sufficient) glutathione reductase (Cao et al., 2020), which likely involve appropriate physiological scavenging of ROS and repair of ROS-induced oxidative damage, and can be interpreted as suggestive of cellular redox involvement. Interestingly, glutathione level declines with age, and the elderly appear to be hit hardest by COVID-19. Iron and ROS can cause abnormalities in fibrinogen cellular behavior (Lipinski and Pretorius, 2012), interpretable as perhaps associated with the observed thrombosis in COVID-19. Additionally, ferroptosis, which is a form of cell death that depends on ROS-centered phospholipid damage and differs from apoptosis, necrosis, and classical autophagy, can be induced by glutathione deficiency and excess iron (Lei et al., 2019). Taken together, these histological and clinical observations in COVID-19 patients and my above-given interpretations point toward a redox/iron/ROS triad as potentially deeply involved in acute COVID-19. Importantly, this triad is strongly interrelated itself because (1) iron transport, utilization as a cofactor, and storage are highly dependent on tight control of its redox state; (2) reductants and iron can be a physiological source of ROS; and (3) reductants can scavenge ROS (Crichton, 2016).

## Cellular Iron, Redox, ROS, Damage, and Repair

A large number of biochemical macromolecules require specific iron coordination for their biochemical function (e.g., oxygen binding and protein-based iron cellular transport). Normally, ~95% of cellular iron is safely bound to various proteins (Crichton, 2016). Hemoglobin is the largest iron utilization protein in the body and, if compromised, can release substantial iron in hemoglobin-centered disorders. Ferritin is the major iron storage protein and is noted to act as an “antioxidant” because it safely sequesters and stores excess iron within its protective shell in an unreactive form. Normally, only a small portion of cellular iron becomes part of a liable, non-protein-bound iron pool (the LIP) and is bound to and carried by molecules such as citrate (Crichton, 2016). Improperly coordinated iron, especially in the presence of high oxygen and reductant concentrations such as in the lung, has the potential to generate ROS such as hydrogen peroxide, superoxide, and the hydroxyl radical (Halliwell and Gutteridge, 1985). The details of iron coordination (i.e., the number and identity of chemical groups bound to the iron donated by endogenous cellular iron carrier molecules or clinical chelators) determine whether the iron is properly coordinated or can produce damaging ROS. In addition to this toxic generation, ROS are also formed cellularly (1) at low concentrations for an intricate network of signaling, (2) for defense against pathogens at higher concentrations [e.g., in the neutrophils by reduced nicotinamide adenine dinucleotide phosphate (NADPH) oxidase], and (3) by enzymes misfiring along normal cycles, although these are expected and generally appropriately handled by the cellular antioxidant systems in healthy individuals. Because hydrogen peroxide is important as a ROS that is active in cellular signaling, excess could be disruptive (Sies, 2017).

There are ~30 enzymes from which ROS can originate, and complicating this further is that both enzymes and improperly coordinated iron can transform one ROS to another potentially increasing or decreasing toxicity or terminating them as water. Superoxide dismutase can convert superoxide to hydrogen peroxide, and catalase can convert hydrogen peroxide to water (Banerjee, 2008). Importantly, one form of a family of glutathione peroxidases can eliminate hydrogen peroxide, and another (GPX4) can repair ROS-damaged phospholipids. Uncontrollable ROS-centered damage to phospholipids is considered a main marker for ferroptosis, and sufficient reduced glutathione level and available GPX4 are crucial in inhibiting and repairing such damage (Lei et al., 2019). Unlike catalase, GPX4 requires the cellular reductant glutathione for enzymatic activity. Glutathione requires NADPH for conversion from its oxidized form (GSSH) to its reduced form (GSH) by the enzyme glutathione reductase, which points to the importance of the availability of the cellular reductant NADPH and even glucose. NADPH, in turn, is synthesized by the pentose phosphate (PPP) pathway from glucose, and general cellular reduction is a marker for metabolic health. Importantly, erythrocytes depend exclusively on this pathway for NADPH availability. Lack of appropriate levels of cellular reductants points toward cellular and oxidative stress and is a predictor of cell death. A compromised PPP through glucose-6-phosphate dehydrogenase mutation and the resulting lack of NADPH production, depending on consumption of certain foods or drugs, can lead to the inability to keep hemoglobin iron ferrous, hemoglobin destruction, iron release, and erythrocyte lysing (Fibach and Rachmilewitz, 2017).

More generally, with respect to ROS-induced cellular damage, the hydroxyl radical causes the greatest by attacking and modifying biomolecules close to it because of its high reactivity and thus low diffusibility. Hydrogen peroxide is considered the least damaging and is able to travel and permeate membranes and superoxide intermediate. ROS cause oxidative damage to cellular constituents including protein side chain modification and polypeptide backbone cleavage, changing their function, stability, solubility, and aggregation profile. Phospholipids are also damaged by ROS, not only changing their function and membrane stability but also producing secondary ROS that go on to cause further cellular damage. Additionally, repair, disposal, and new synthesis of unrecoverable proteins and phospholipids that are initiated by ROS-induced damage are taxing to cells both materially and energetically (Muhoberac and Vidal, 2019). This iron-centered ROS damage detracts from other infection-fighting cellular responses, weakening the cell and disrupting physiology.

## Chemistry of Iron, Chelators, Reductants, and ROS

Iron behavior differs substantially from atoms that do not undergo redox chemistry. Ferrous and ferric iron have different aqueous solubilities, preferences for coordinating groups, and reactivities. Binding constants for ferric can be several orders of magnitude greater than ferrous for the same bound molecule. Iron coordination alters the ability of iron to be reduced whether in aqueous solution or in the cell. Furthermore, bound molecules may transiently dissociate as part of normal chemical equilibrium or, more permanently, as composition and concentrations change (e.g., during disease progression). Ascorbate and GSH can both coordinate and reduce iron, and these molecules when in the cell are part of its overall redox balance. *Importantly, the interplay between iron and cellular iron-binding molecules and reductants in the LIP is perhaps more likely to enhance the production of ROS under pathological conditions*. Finally, the binding of exogenous iron chelators, as might be used in therapeutic treatment, must be considered. Chelation is a general term referring to a single metal-binding molecule that has more than one metal-binding, coordinating group, and as such, a chelator may also be endogenous (i.e., citrate, or a drug).

Iron-centered ROS chemistry is generally introduced and explained by examining the generation of toxic hydroxyl radicals in terms of the Fenton/Haber Weiss-type reaction, by considering the reactions in a solution of ferric iron salt and hydrogen peroxide with no chelating molecules and no specific reductant added. However, this approach is somewhat less in parallel biologically with an ROS generating system that would contain cellular-like iron chelation of some form and a directly available reductant (Muhoberac and Vidal, 2019). Such conditions are supplied by Udenfriend's reagent, which generates hydroxyl radicals and contains iron, ethylenediaminetetraacetic acid (EDTA), and ascorbate, such that (1) the iron is subject to coordination by carboxylates and nitrogen donors from EDTA, and (2) the available reductant parallels that of the cell. Interestingly, ascorbate can be replaced by other cellularly relevant compounds such as thiols as reductants in the Udenfriend system enhancing hydroxyl radical generation (Nappi and Vass, 1997). In normal cells, both ascorbate and GSH concentrations can be quite high—in the millimolar (mM) range. Additionally, EDTA, because of constraints in its chemical structure, has an iron coordination site open to improper coordination by small molecules (e.g., dioxygen or hydrogen peroxide) for ROS-generating reactions or transformations.

Using clinical chelators, as examples, Desferrioxamine is a hexadentate binder providing six iron coordination bonds, whereas Deferiprone (bidentate) can supply a maximum of two and would thus require a binding ratio of 1:3 iron/Deferiprone to completely saturate iron in its normal octahedral coordination. Clearly, the cellular availability of the latter needs to be higher for complete iron binding saturation, which turns out to be very important. If less, iron would be open to interact directly with small molecules and perhaps a reductant like ascorbate. This situation, which can be considered improperly coordinated iron, is dangerous in that it allows generation of or conversion between ROS, as mentioned earlier. Studies show that bound hexadentate Desferrioxamine is not likely to generate ROS, but Deferiprone can do so at low concentrations in that hydroxyl radicals from Deferiprone are formed at 1:1 and 1:2 solution ratios but are greatly attenuated at a higher ratio (Devanur et al., 2008). Furthermore, there may exist competition between the clinical chelator and available cellular binding molecules potentially leading to mixed iron coordination and making predictability difficult. *Excess iron removal is clearly important, but choosing the proper clinical chelator and concentration that does not increase ROS production is a more important consideration*.

Important contributions to the complexity of iron behavior in cells are the variety and concentration of endogenous iron binding molecules normally found in the LIP. These include both redox active and non-active molecules. Generally, citrate and glutathione are mentioned as the most likely endogenous iron binders; however, the cellular milieu has many others with appropriate groups for iron coordination (e.g., lactate, phosphate, and ascorbate). Both GSH and ascorbate can reduce iron and enhance hydroxyl radical production *in vitro*, which leads to a curious situation. How can these two reductants, which are well-known as “antioxidants,” have a dual role of producing damaging ROS? It turns out that both reductants have pro- and antioxidant ability. In the Udenfriend system of hydroxyl radical generation, low concentration of ascorbate enhances hydroxyl radical production through reducing the iron, but at higher concentration, ascorbate has the ability to scavenge the radicals it helps produce (Miller and Aust, 1989; Griffiths and Lunec, 2001). Similarly, GSH can enhance production of these radicals at lower concentration but apparently scavenges them as GSH concentration is increased. Although these higher ascorbate and GSH concentrations for ROS scavenging are not unrealistic physiologically, they reduce with age and disease. *This dual role points toward the need for maintaining (1) high physiological concentrations of reductants and their regeneration (re-reducing) systems for clinical efficacy and (2) availability of synthesis precursors (e.g., cysteine for GSH)*. Furthermore, this concentration dependence for efficacy recalls the debate on utility of ascorbic acid to treat a variety of conditions (e.g., sepsis, where a high ascorbate concentration may be the determining factor). In addition, the ascorbate and GSH cellular pools are linked through their interconversion and through NADPH-dependent regeneration producing GSH from its oxidized counterpart, GSSH, as mentioned earlier. A different kind of dual behavior is found with some clinical chelators. Desferrioxamine is capable of scavenging hydroxyl radicals in an efficient manner, increasing with additional chelator concentration (Kayyali et al., 1998). This ability is independent of iron and only refers to the trapping of radicals by the molecular structure of Desferrioxamine itself. Deferiprone appears to be less capable of such direct trapping. Thus, in addition to the ability of a chelator to properly coordinate iron (i.e., bind and shield iron from dioxygen or ROS interaction), the intrinsic ability of a chelator to scavenge radicals may be important to consider. In fact, this may eventually be designed into the chelator, or perhaps, ROS scavengers could be administered separately.

## Pathways of ROS Damage to Proteins, Lipids, and Cells

Examining the triad of depressed cellular redox, elevated iron, and ROS-induced damage, what are the potential mechanisms that may contribute to COVID-19? First, pulmonary cells are particularly susceptible to oxidative stress under normal conditions, and both their epithelial lining and lavage normally show high concentrations of the antioxidants GSH and ascorbate. Under ARDS, both total and non-heme iron are increased, markers for ROS-induced damage of proteins and lipids are found, and there are decreases in ascorbate and GSH (Chabot et al., 1998). Second, there are well-known, non-COVID-19, potentially life-threatening hemoglobin-related conditions associated with elevated iron and enhanced cellular oxidative damage and stress. Hemoglobin must contain iron-porphyrin (heme) in the ferrous state for oxygen binding and transport, even though hemoglobin normally undergoes spontaneous oxidation at a few percent a day, releasing damaging superoxide radicals. An uncompromised erythrocyte can deal appropriately with this through phospholipid repair enzymes and direct ROS scavenging by GSH, and through a NADPH-dependent enzyme, methemoglobin reductase, which re-reduces the iron (Fibach and Rachmilewitz, 2017). Still, ferric iron in and ROS damage to hemoglobin contribute to Heinz body formation and cellular lysing, and heme released from hemoglobin can produce ROS. Importantly, both GSH regeneration and the reductase require availability of the reductant NADPH, and with COVID-19-compromized erythrocytes, this may be problematic because of cellular damage, overburdened pathways, and lack of available precursors. Third, a direct thrombolytic condition may arise from improperly coordinated, excess iron release and its interaction with fibrinogen. Iron directly interacts with fibrinogen causing an accelerated form of coagulation that is not ordered structurally like fibrin and is resistant to dissociation with sodium dodecyl sulfate (SDS) and proteolysis (Lipinski and Pretorius, 2012). Fourth, the cell death process of ferroptosis is directly dependent on iron and ROS in that iron chelators can halt the process and insufficient GSH levels accelerate it. Interestingly, one of the markers for ferroptosis is ROS-induced phospholipid damage as found in ARDS. In ferroptosis, phospholipids are repaired by the selenium-containing enzyme GPX4, which requires GSH for its function. This enzyme-based lipid repair is in addition to the direct scavenging and elimination of ROS by GSH, if available. As mentioned earlier, COVID-19 survival has connections to higher selenium level and non-elevated glutathione reductase, which suggest sufficient repair enzyme and GSH availability. Fifth, GSH availability hinders not only ROS damage, which can lead to direct protein crosslinking, but also crosslinking by keeping sulfhydrals reduced. Such crosslinking and ROS damage contribute to protein unfolding and aggregation, which must be handled by a complex degradation system further stressing the cells. In addition, aggregated or denatured protein may produce sites of improper iron coordination-generating ROS. Finally, even the structure and binding behavior of CRP are altered by ROS modification (Singh et al., 2017), which may lead to an additional deleterious cascade of events.

## Conclusion: Enhanced Reduction and Chelation for COVID-19

The redox/iron/ROS triad of COVID-19 is complex and itself highly interrelated and does not address directly other ongoing viral-related degrative physiological processes. Such processes have a number of more standard treatment approaches ranging from antivirals to immune system modulators. However, the alteration in cellular redox balance and the need to repair and eliminate damaged proteins and membranes stemming from this COVID-19 triad are clearly taxing to the cells and detrimental to the execution of cellular antiviral defenses, let alone general metabolism, and these are likely important components of the overall disease mechanism. *The two most fundamental approaches to therapeutic interventions that would ameliorate the negative effects of the redox/iron/ROS triad would be to reduce improperly coordinated iron levels and enhance reductant levels substantially, particularly in severe COVID-19 cases*.

The purpose of my perspective is not to be prescriptive but to elicit open mindedness in the exploration of options for COVID-19 treatment. Clinical iron chelators are available, and reductants, including ascorbate and GSH, have been used as IV treatments for a variety of conditions ranging from septic shock through viral diseases to ischemic stroke. NADPH is a precursor reductant relative to GSH in that it is used directly for reduction in GSSH to GSH by glutathione reductase, and GSH can reduce ascorbate. However, GSH and ascorbate can be immediately available through IV administration, not requiring enzymatic transformation. Additionally, ferric iron is reduced to ferrous in hemoglobin by methemoglobin reductase, ascorbate and GSH, which rescues hemoglobin for dioxygen binding, and a hallmark of COVID-19 is problematic oxygen distribution. Proper choice of chelator and performing sequential administration of chelator then reductant may be important in preventing initial ROS formation enhancement. Finally, a substantial increase in the reductant levels may be necessary for overwhelming, direct scavenging of ROS. Taken together, clinically treating the redox/iron/ROS aspect of COVID-19 in this manner is likely to be an important positive contribution to the disease outcome and perhaps a dominant contribution especially over some portion of disease development timeframe.

*It should be noted that the pharmacological options being discussed herein are a combination of ideas stemming from in vitro and in vivo research and thus are theoretical. They are put forward here by a non-clinician and should only be considered in that light*. Furthermore, since the submission of this manuscript 3 months ago, publications addressing separate aspects of the redox/iron/ROS triad have begun to appear in the literature. To my knowledge, these reports do not treat redox, iron, and ROS in a unified manner as is done in this perspective, nor do they discuss many other details treated herein. These publications are similar to occasional articles over the last decades suggesting using reductants to treat various diseases.

## Author Contributions

The author confirms being the sole contributor to this work and has approved it for publication.

## Conflict of Interest

The author declares that the research was conducted in the absence of any commercial or financial relationships that could be construed as a potential conflict of interest.
